# Nano-Structured Carbon: Its Synthesis from Renewable Agricultural Sources and Important Applications

**DOI:** 10.3390/ma15113969

**Published:** 2022-06-02

**Authors:** Harishchandra Jirimali, Jyoti Singh, Rajamouli Boddula, Jung-Kul Lee, Vijay Singh

**Affiliations:** 1 Tarsadia Institute of Chemical Sciences, Uka Tarsadia University, Maliba Campus, Gopal-Vidya Nagar, Surat 394350, Gujarat, India; hdj739@gmail.com (H.J.); jyotisingh7july@gmail.com (J.S.); rajamouliboddula@gmail.com (R.B.); 2 Department of Chemical Engineering, Konkuk University, Seoul 05029, Korea

**Keywords:** agricultural waste, nano-structured carbon, methods of synthesis

## Abstract

Carbon materials are versatile in nature due to their unique and modifiable surface and ease of production. Nanostructured carbon materials are gaining importance due to their high surface area for application in the energy, biotechnology, biomedical, and environmental fields. According to their structures, carbon allotropes are classified as carbon nanodots, carbon nanoparticles, graphene, oxide, carbon nanotubes, and fullerenes. They are synthesized via several methods, including pyrolysis, microwave method, hydrothermal synthesis, and chemical vapor deposition, and the use of renewable and cheaper agricultural feedstocks and reactants is increasing for reducing cost and simplifying production. This review explores the nanostructured carbon detailed investigation of sources and their relevant reports. Many of the renewable sources are covered as focused here, such as sugar cane waste, pineapple, its solid biomass, rise husk, date palm, nicotine tabacum stems, lapsi seed stone, rubber-seed shell, coconut shell, and orange peels. The main focus of this work is on the various methods used to synthesize these carbon materials from agricultural waste materials, and their important applications for energy storage devices, optoelectronics, biosensors, and polymer coatings.

## 1. Introduction

Carbon is an attractive atom in several fields, and it can form a bond with different small atoms to make many compounds together with other carbon atoms, leading to the formation of stable covalent bonds. Carbon is weakly reactive in comparison to different elements underneath regular conditions. It resists oxidation and it does not react with sulfuric acid, hydrochloric acid, chlorine, or any alkali metals at high temperature and pressure. Carbon will react with oxygen to form carbon oxides at high temperatures and metals to form metal carbides. Carbon has a large flexible electronic structure and is the essential element for improving carbon skeleton-based chemistry. Carbon from many allotropes, such as graphite and diamond, is a photophysical example of allotropic carbon where it exists in several allotropic forms in nature. In addition, all the allotropes exhibit an extensive variety of physical properties. Among the classes of nonaromatic systems, diamond is important for its carbon-containing sp^3^ hybridized. It has the least abrasive material of diamond and may lead to being an electrical insulator and thermal conductor. Graphite is one of the softest recognized materials, with sp^2^ hybridization and graphene sheets organized in layers affording its soft internal nature. Contrarily, graphite is opaque, a superb lubricant, a great conductor of energy, and a thermal insulator. Carbon allotropes are not restrained to diamond and graphite, but also include Buckballs (fullerenes), amorphous carbon, glassy carbon, carbon nanofoam, and nanotubes [1,2]. For further details, the reader can be referred to excellent reviews concerning the carbon-based applications and their precursors’ production [3,4,5].

Carbon nanoparticles are used in carbon quantum dots (CQDs) that are the latest class of carbon nanoparticles and have a massive luminescence with particle sizes smaller than 10 nm, normally functionalized in corporations of hydroxy (–OH), acid (-COOH), thio (-SH), and amine (–NH_2_), etc. Nanocarbon has attracted attention because of its eminent properties and received much research attention due to its low toxicity, desirable biocompatibility, substantial synthesis of raw substances, and low fabrication cost. Few of the literature suggests the cost of the materials where these renewable sources are placed, and notable observations found that these materials are a quite reasonable cost [6,7,8,9,10]. Various nanoforms of carbon show tunable emission spectra, good photoluminescence stability, resistance to picture bleaching, large two-photon absorption area, and smooth functionalization [11,12,13,14,15]. In addition, metal–organic framework (MOF)-derived nanoporous carbons are interesting and have a very high surface area so they can be interesting to the agricultural aims [16,17]. In addition, they have attractive controllable porosity, thermal and chemical stability, catalytic activity, and high electrical conductivity. Several approaches have been reported for MOF-derived carbon structures, such as hetero atom doping, hybridization parameters, precursor control, etc. [18]. Recently, MOF-based nonporous carbon reported with cation exchange membrane and compatible nanofiller to fabricate defect free matrix pervaporation membranes were discussed [19,20,21].

Due to their eminent properties, nanocarbon-based materials are attractive and used as a promising material for wide applications in various fields such as medicine, biotechnologies, and biomaterials. In biotechnology, carbon nanoparticles are used in the preparation of biosensors, bio-stimulators, and micropatterned surfaces for the creation of microarrays for genomics and proteomics. These carbon-based materials comprise nanocomposite polymer films, amorphous carbon, pyrolytic graphite, nanocrystalline diamond films, fullerenes layers, and single and multi-walled carbon nanotubes. Polymer/nanocomposites draw attention to the fields of automotive, aerospace, construction, and electronic industries due to their unique properties, which make them substantial candidates for these applications [22]. Increasing greenhouse gas emissions suggests the ban or controlled use of carbon-based fuels which are emitting a considerable amount of carbon dioxide. The produced energy is replacement by hydro and solar power can be stored in batteries and used for automobile applications via electrically driven hybrid vehicles. It is mandatory towards high claim from the market area for new hybrid applications and high-performance capacitors. In addition, it requires a high-power device to provide start-up and to change acceleration energy allied with converting into useful electricity [23,24,25]. Recently, supercapacitors in the form of electrochemical double layer capacitors (EDLCs) are mainly carbon-based due to their low cost, high electrical conductivity, good chemical stability, and impressive lifespan [26]. In view of the current developing industries, as well for domestic applications, fulfilling the energy requirement requires highly efficient energy storage devices with high surface area carbon to produce the energy storage devices. For the development of advanced energy storage devices such as batteries, supercapacitors, flow cells, and electrode fields need high-performance electrodes, and carbon with nanostructure and high surface area is the best option [27,28,29,30]. Compared to other nano-families, carbon nanostructures are extensively used and have attracted strong research attention [31,32]. The properties of carbon nanostructures are improved by combining to form certain materials which can be useful in the devices. The combining of carbon annotates, fullerenes, and graphene oxide can enhance the properties due to the formation of a porous structure and conductivity. The nanocarbon also gives improved properties with the combination of the carbon nanostructures [33]. 

Hierarchical cellulose nanowhiskers (CNW) display a unique, multi-scale, porous shape made from micro-and mesoporous linked by means of tubular macro-pores, giving better surface location and floor accessibility [34]. The nanocarbon structure consists of the graphitic structure of all carbon nanotubes (CNTs) and its morphology is distinct from traditional multiwall CNTs, with exclusive wall morphology and well-ordered graphitic layers [35,36,37]. New materials, including carbon fibers, glass-like carbons, pyrolytic carbons, and so forth, have been found widely in industrial packaging. Lithium-ion rechargeable batteries are the latest application of this carbon material that uses nanostructure carbon anodes to make viable portable digital devices. The nanometer-scale texture based on the preferred orientation of anisotropic hexagonal layers plays a critical role in their qualities [38]. Several new graphitic materials are used in nanostructural devices within a complicated hierarchical shape, together with, for instance, carbon fibers which include carbon nanotubes in their cores [39]. Along with these materials, some boron-doped diamonds are utilized in wastewater remedies with the aid of the electrocatalytic oxidation performance of organic pollutants that depend strongly on the electrode substances [32]. The normally used electrode materials include Ti/PbO_2_, Ti/RuO_2_, Ti/SnO_2_ (Ti = titanium, Pb = lead, Ru = ruthenium, Sn = tin), and boron-doped diamond (BDD) electrodes [40,41,42,43]. Among them, BDD electrodes have attracted sizeable attention due to their many desirable properties, including appropriate physical and chemical balance, high oxygen evolution potential, and a huge electrochemical capacity window [44,45].

BDD electrodes can produce large amounts of distinctly reactive heterogeneous OH on the electrode floor, owing to excessive oxidation and mineralization strength within the remedy of natural pollutants. Furthermore, OH is changed into a powerful oxidant in the oxidation process [46,47,48,49]. Carbon consists of allotropes, such as graphene made by using mechanical exfoliation from graphite. Due to attracted assets of graphite oxide, researchers have explored less complicated, more efficient, less expensive, and better yielding strategies of synthesizing graphene, which may be scaled up without difficulty and preserve commercially visible qualities for industrial application [49]. Graphite is oxidized with strong oxidizing chemicals to deliver oxygenated functionalities within the graphite structure, which makes the material hydrophilic and also expands the layer separation. This selection makes it feasible to exfoliate the graphite oxide in water through sonication which finally produces a monolayer of oxygen functionalized graphene with a few layers, known as graphene or graphene oxide (GO). 

In addition, graphene oxide and graphite oxide differ in terms of their layer form. Even though graphite oxide is a multilayer system, it also contains monolayer flakes and few-layered flakes. Graphene oxide clumps may be removed from the water for disposal, and can also act as the sensor which could locate a completely low level of most cancer cells. The level might be as low as 3 to 5 cancer cells in one milliliter of blood. Graphene oxide has a porous structure and binds very easily to the antibodies that attach to the cancer cells. The main cancer cells are then marked with fluorescent molecules to highlight them in a microscope. Graphene oxide can be used as an electrically insulating layer wherein conductive graphene nano wires can be used. If heat is applied through atomic force microscopy, oxygen molecules on graphene oxide are isolated to afford conductive nanowires. The nanostructure carbon field is attracting extensive research in nanotechnology, with swift advances being made [49].

## 2. Useful Methods for the Synthesis of Nanostructure Carbon from Agro-Waste

**Figure 1:**
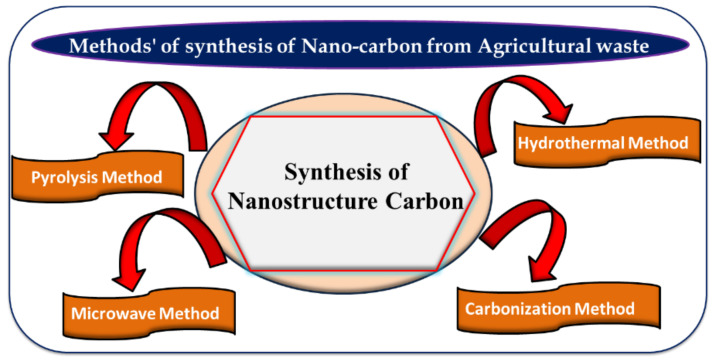
Various methods such as pyrolysis, microwave, hydrothermal, and carbonization for nanocarbon synthesis from agricultural waste.

Several natural methods are known for making carbon from agricultural waste, such as incomplete burning of wood and agricultural waste, but this causes enormous environmental pollution due to carbon dioxide emissions [50]. There are scientific methods for the conversion of biomass into carbon nanomaterials to enable the tuning of different structural and functional properties [51]. Bio-inspired methods are also used with natural biological materials for the systematic production of nanostructured carbon materials [52]. According to the application of the nanocarbon, their structures such as pore size, surface area, chemical composition, and functional groups can be tuned by varying the synthesis conditions [53,54,55]. Reduction of the graphene to graphene oxide nanosheets was prepared with Hammer’s method to receive high purity graphite, and reported being cost effective, eco-friendly, and efficient [56]. Synthesis of carbon nanostructure by agro-waste was reported with well-known methods and their CNTs materials were described [51,57]. Several methods are listed here for the synthesis of nanostructured carbon from renewable agro-waste (Figure 1).

### 2.1. Pyrolysis

**Figure 2:**
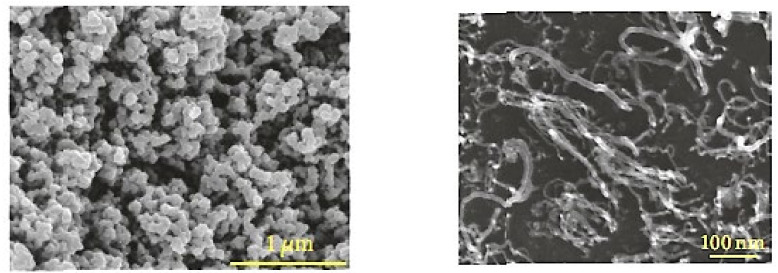
Carbon nanostructure growth, (**Left**) Spherical structures, (**Right**) Tubular structure. Adapted with permission from Ref. [61].

Among the various methods for nanostructured carbon synthesis, pyrolysis is a simple and popular thermochemical method that disintegrates the carbon sources into smaller components in the absence of oxygen. Traditionally, this method is used for the fractionation of petroleum products, but now pyrolysis is gaining attention due to its modifiable renewed process for the renovation of wastage agricultural products into economically and environmentally beneficial products [58,59,60,61]. Figure 2 shows the carbon nanostructure with spherical and tubular structures.

Pyrolysis uses carbon dioxide and a mixture of pyrolytic gases such as nitrogen, methane, hydrogen, and carbon dioxide in a tubular furnace at a high temperature of more than 500 °C. In this method, stalks of wood or agricultural waste were ground into powder to reduce the particle size. The tubular reactor uses purely ground quartz silica in the form of a tube with a small diameter of around 2.5 cm and a length of around 60 cm. One vacuum chamber can be used for the creation of the vacuum in the tube. Ground agro-stalks powder used for the preparation of the carbon is placed in the tube, and at high temperature, the nitrogen and carbon dioxide gases are introduced into the tube furnace and then collected after the biochar is formed [58]. Steam pyrolysis uses steam at higher temperatures. The powdered agricultural waste materials are fed into the quartz tube furnace. The steam is generated separately in the round bottom flask on the heating mantle and directly connected to the pyrolytic furnace tube. The furnace is heated to 600 to 700 °C in the presence of steam which produces gases that are expelled with the steam, thereby avoiding overheating the carbon material. The steam also preserves the natural porosity of the waste materials used for the carbon preparation [62].

In spray pyrolysis, the waste materials are sprayed directly into the pyrolytic furnace at a high temperature to form carbon particles. Nebulized spray pyrolysis is a subtype of spray pyrolysis where the reactant and catalysts, such as waste cooking oil and ferrocene, are added and mixed in the nebulizer. The reactants in the quartz tube were nebulized and the tube was placed in the pyrolytic furnace with a flow of inert nitrogen gas. In the presence of nitrogen and high temperature, the carbon is carbonized to form the nanostructured material [63,64]. Ultra-sonication is a technique where ultrasonic waves are to activate the molecules and also disperse the materials. The ultra-sonication device is used to generate the spray in the spray pyrolytic process to form fine droplets directly into the quartz furnace connected to the spray chamber and placed inside the furnace. This method combines economical and green sources by avoiding the sacrificial catalytic template. Carbohydrates and sodium bicarbonates in an aqueous medium are treated with ultra-sonication after conversion by being sprayed in a previously heat-activated tubular reactor. In the inert and hot environment, the sprayed solvent is vaporized to leave the residue of the precursors which are immediately decomposed at high temperatures. The use of sodium bicarbonate and carbonates as the reactants produces bubbles on the posterior part of the furnace, which plays an important role in the material formation. Decomposition of sucrose occurs at a high temperature, which arises in spray pyrolysis. Bicarbonate salts act as a catalyst and the gases produced during the process form the porosity in the carbon [65,66]. Activated carbon is one of the forms of the nanostructure carbon that can also be synthesized by a thermochemical method where KOH is added as an activator. For the synthesis of the activated carbon from the agro-waste, the dried parts of the bamboo stem and coconut shells were ground to powder and mixed with KOH at 1:3 ratio and boiled in a water bath for a specified period. The samples were washed to remove the salts and placed in a stainless-steel vertical tubular reactor. The pyrolysis was carried out at 800 °C for the specified period and the heating was discontinued. The cooled product was washed with deionized water and again kept in the oven at 100 °C for drying [67].

### 2.2. Microwave

The microwave irradiation method for the synthesis of carbon materials is important because of its fast process and low energy consumption at high heating in a closed chamber at different energy modes, using the irradiation frequency to finely control the temperature and time. Different nanostructures with controlled porosity and shapes can be obtained. The micro cellulose obtained from the agricultural waste products has applications in the synthesis of porous carbon materials by microwave irradiation. The solution of the microcrystalline cellulose and a polymer was added to a glass and subject to microwave irradiation to form the solution that then underwent microwave annealing after irradiation to form the microporous carbon sponge [68]. Waste palm was converted into the powder form and placed for the carbonization at different temperatures and obtained carbon, which was mixed with KOH and kept in the microwave for the activation to increase the capacity [69]. The palm oil is removed from the kernel waste and collected then ground to make powder, which is placed in a microwave pyrolysis chamber at 1000 W and 2.45 MHz in a nitrogen inert atmosphere for about 30 min to generate porous carbon. The temperature variation is measured by the infra-red thermometer [70].

Domestic microwaves have also been used for the production of biochar from waste palm oil kernels by simple microwave irradiation in nitrogen purging [71]. Different parameters affect the physical structure and chemical properties of the carbon, including microwave irradiation time, microwave frequency, microwave power, and impregnation ratio [72]. A fluorescent carbon dot was also synthesized by microwave pyrolysis method from natural sesame seeds that were collected, cleaned, and placed in a domestic microwave. At 800 W microwaves power, the seeds were carbonized by irradiation, and the resulting sesame seed oil was separated out to form a carbonized residue. The agglomerates in this carbonized residue are separated and removed by centrifuging at 8000 RPM residue. The remaining material is lyophilized to give green fluorescent carbon dots. This is a green method for the synthesis of fluorescent carbon from natural sources [73]. For a comparative study of the microwave and conventional pyrolysis methods, activated carbon was prepared by both methods, its characteristics are analyzed, and the results demonstrate that the carbon prepared by microwave and pyrolysis has similar characteristics and which was synthesized from sesame husk shells [74]. 

### 2.3. Hydrothermal

The hydrothermal process is a natural process for the conversion of buried biomass to coal, gasses, and liquefied natural oils. Nowadays, this method is used for the conversion of agricultural waste products to fuels and carbon nano materials. Hydrothermal processes are generally carried out in the presence of water at high pressure. This process is economical and green as water is the solvent which, upon heating, produces the water vapors to create a high pressure inside the closed chamber. The HTC goes through three steps, such as material dehydration to produce the furfural derivatives, followed by polymerization of the modified product, and finally dehydration of the produced material to form the final carbon [75]. The waste materials such as banana peels were collected, washed, dried, ground to fine homogeneous particle size, and placed in a hydrothermal reactor at 80 °C for six hours. The obtained product was repeatedly washed and collected by centrifugation and dried in an oven to produce the banana-peel carbon [76]. The waste materials consist of most of the hydrocarbons which contain natural carbohydrates and polymers, including cellulose and lignin. Different parameters can be applied for the production and the physiochemical properties of the carbon can be tailored by varying the temperature, catalyst contact time, and substrate concentration [77]. Grape seeds, the waste materials of the viniculture industry, were again used for oil production and the residue remaining after the removal of oil was used for the production of carbon. This residue was washed and collected as a powder and placed in a jacketed batch autoclave reactor. The carbon properties can be tuned by varying the temperature [78]. Hydrothermal carbonization was carried out in a stainless-steel reactor, and sometimes Teflon beakers were used inside the chamber. Higher temperatures approaching the critical temperature, where the dielectric constant decreases and hydrogen bonding starts to break, produce hydronium ions and hydroxyl ions. Sometimes this reaction can produce hydrocarbons which can act as an acid catalyst [79]. The residue has also been converted to hydrochar by hydrothermal treatment, where the residue is autoclaved at different temperatures and times to produce high- and low-density carbon. The hydrochar produced in the first step has numerous oxygen functionalities and its pyrolysis removes the water by the process of condensation and produces carbon. Activated carbon can also be prepared by mixing acidic or alkaline materials in the reactor [80].

### 2.4. Microwave Hydro-Thermal Carbonization (MHC)

In this method, microwave energy is used to heat the hydrothermal unit in which carbonization occurs. This method is faster than the normal hydrothermal method, as microwave heating is an efficient, economical, and fast way to induce the reaction. Rice straw is collected, cleaned, chopped, and ground to make homogeneously sized straw dust, which is placed in the digestion tubes, and then water is added. Digestion units are kept in the microwave and the power is adjusted up to 1200 W and the temperature to 230 °C by varying the microwave parameters. The produced hydrochar has different physicochemical characteristics according to the temperature, biomass to water ratio, particle size of straw, and reaction time. The hydrochar can also be prepared from the rice husk, which is washed, cleaned, dried, and ground to make the powder which is placed in the hydrothermal unit under microwave irradiation [81,82]. 

### 2.5. Carbon by General Carbonizations

In this method, the carbonization of the agricultural waste was achieved in an open fire by simple chemical pre-treatment or using simply closed containers. Rice husk, wheat straw, and corn cabs were collected, washed, and dried in an oven and ground to make powder. The chemical activation is achieved by mixing sodium chloride with the obtained powder at the ratio of 4:1 Sodium Chloride: powder. The mixture was placed in an aluminum container with five openings for gas exhaust and kept in an open wood fire for 30 min for carbonization, after which the activated material was removed, washed with distilled water, and dried [83]. The corn cob was carbonized in a kiln with a drum length of 90 cm and diameter of 60 cm. The biomass was fed from the bottom and initially the drum was kept open at the top gas exhaust. Carbonization occurs in the low oxygen environment and the carbonized product was used in the preparation of briquettes [84]. The Elsa barrel technique was used for the production of biochar from agricultural waste such as rice husk, saw dust, groundnut husk corn cobs, and cassava. The biomass was cleaned, chopped, dried, and ground, and the Elsa barrel was packed with the biomass. At the open end, the fluid fuel was added and ignited. The open end of the barrel was closed, a chimney was added because of incomplete burning occurring due to the low oxygen environment, and biochar was produced [85]. Graphene oxide was prepared from sugarcane bagasse, which remains after the extraction of the juice from sugarcane. The residue was collected, dried, and ground to make the powder, which was mixed with ferrocene and kept in a muffle furnace in the crucible at 300 °C for 10 min. The produced black solid was collected and the formation of graphene oxide in the product was confirmed [86]. In the dry season, natural firing occurs in forests with a variety of trees. Samples of two different sites of the matured pine trees were collected and analyzed to confirm the occurrence of CNT and the presence of graphitic carbon in the collected samples. Graphitic particles are created in the natural burning of Texas pennon pine wood chips in the air, and they were collected on the glass wool filters using the thermophoretic precipitation method. The analysis of the samples confirmed the formation of the CNTs in the burnt wood samples [87,88]. 

## 3. Preparation of Nanocarbon from Various Agricultural Wastes

### 3.1. Sugarcane Waste

Preparation of nanocarbon from sugarcane bagasse (SCBAC) is a potential approach to developing low-cost and competent adsorbents for fuel pollution removal. It is available at low cost, with abundance as a renewable resource. Few reports suggest the biochar yields are different at different temperatures of preparation. The calculated analysis of the sugar cane bagasse and rice straw prepared biochar is 0.93 and 0.79 USD/Kg, respectively. It clearly indicates the waste resources are effectively working as a renewable, which can be more feasible to reduce the cost [89,90]. Many naturally available sources have been used as a source for nanocarbons. Pyrolysis of sugar cane bagasse is a novel technique for the production of carbon nanomaterials. The source materials are collected and washed with hot deionized water, dried at 110 °C for two days, and ground in a mechanical curler mill to afford nano-sized particles via two steps: carbonization of the raw material and activation of the ensuing char at an increased temperature in the presence of suitable oxidizing gases, along with carbon dioxide, steam, air, or their mixtures.

Activation of sugarcane bagasse char: For this, 10–15 g of sugarcane bagasse char was loaded into the furnace reactor, and 5–10 sugarcane bagasse chars emerge from the reactor situated within the furnace. Then it was heated at a heating speed of 20 °C/min to a carbonization temperature of 800 °C under an inert atmosphere. After activation, the obtained hard product was cooled to room temperature.

**Figure 3:**
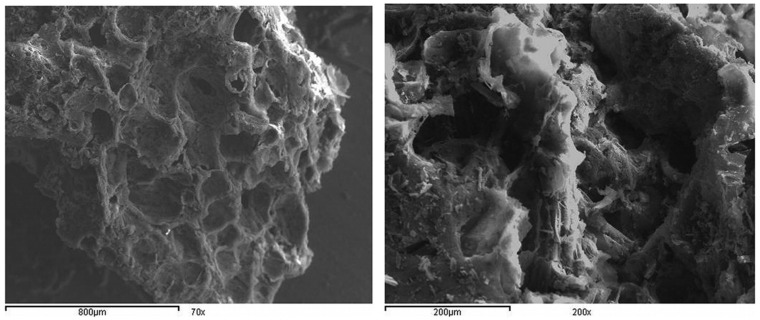
SEM image of SCBAC with 70×, 200× (**left**), and 1500× (**right**). Adapted with permission from Ref. [92].

Characterization of nanocarbon: By using a surface analyzer, the porosity of the nanocarbon samples (Figure 3) was determined under inert atmospheric conditions at −190 °C [91,92]. Chemical activation process: Waste materials were composed and cleaned with deionized H_2_O and dried in a hot oven at 120 °C for one day, then ground, sieved, and transferred to a muffle furnace at 500 °C under atmospheric air for two hr to remove some oxygen and gaseous hydrogen with nanocarbon elements, while the remaining carbon atoms form a glowing crystallographic morphology known as elementary graphitic crystallites. Carbonization usually enhances surface activity and lowers the adsorption capacity. The prepared nanocarbon is furthered washed with a 30% aqueous solution of ortho-phosphoric acid at 1:1 ratio for one day and then washed many times with hot deionized H_2_O [93].

### 3.2. Pineapple

Ananas comosus is an attractive substitute potential source of nanocarbon as it is the cheapest and most easily available raw material. It produces nanocarbon with high adsorptive activity and excellent chemical and thermal constancy, and also high reactivity [81].

#### 3.2.1. From Pineapple Solid Biomass

Nanocarbon from pineapple solid biomass is prepared via the activation method. The solid raw material is composed and rinsed with hot deionized H_2_O and dried in an oven at 110 °C. These dried samples are cut into small pieces with irregular sizes, then further transferred into a 500 mL beaker containing zinc chloride (activation agent, 136.28 g/mol, at a ratio of 1:1, pineapple waste biomass: ZnCl_2_) and immersed for two days at ambient temperature in a magnetic stirrer at constant stirring. It is then dried at 100 °C for one day followed by carbonization at 500 °C for 1 h. After cooling, this sample is washed with cooled distilled water to remove dissolved impurities of ZnCl_2_ and dried at 100 °C for 24 h prior to further use [94].

#### 3.2.2. From Pineapple Crown

Many nanocarbons were prepared through the physical activation process. The collected waste raw material was rinsed with deionized water and dried in the presence of sunlight for 2–3 days. The subsequent pre-carbonization involves heating the pineapple crown waste at 50 °C to 250 °C for 3 days and milling to give a smooth, fine powdered blackish precarbonized product [95,96,97]. This fine powder is activated by potassium hydroxide and converted via single-step pyrolysis to monolith by using a hydraulics machine [98,99]. Further steps involve single step pyrolysis and involve temperature from 30 °C to 500 °C under an inert atmosphere. In this process, temperature is raised constantly [100].

### 3.3. Risk Husk

**Figure 4:**
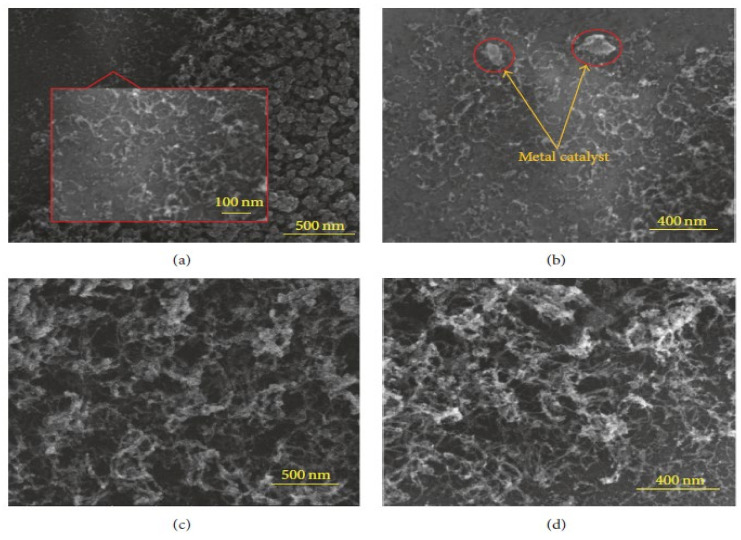
FESEM images of the RH derived CNTs by MO technique for 38 min with (**a**) 500 nm, (**b**) 400 nm (weak interaction between RH and substrate), (**c**) 500 nm, and (**d**) 400 nm. Adapted with permission from Ref. [61].

The microwave oven (MO) method was used to synthesize nanocarbon with the help of paddy, referred to as rice husk (RH). RH is an economically excellent precursor of carbon. In this process, RH is collected, washed with distilled H_2_O using sonication, and further dried at 65 °C for one day. The dried material is ground into fine powder in a mechanical grinder and sieved. Ferrocene is used as a catalyst in the presence of polar solvent (ethanol). After being sonicated for 20 min at room temperature, 120 mg of the dried RH powder and 80 mg of Fe(C_5_H_5_)_2_ solution are mixed and placed on 2.5 cm × 2.5 cm aluminium sheet as the substrate (Figure 4) [61]. The aluminium casing is used to cover the aluminum sheets. The sample is transferred into a quartz tube within a microwave oven (600 W, 2.45 GHz frequency). After plasma processing, the collected sample is cooled at room temperature [101,102]. 

In most cases, RH is first washed with 5–10% HCl and then with deionized H_2_O several more times. After being hot oven-dried at 100 °C for six hours, it is pyrolyzed in a muffle furnace at 300 °C for 3 h and the product is ground into small pieces to give rice husk char (RHC). Finally, RHC is leached out with sodium hydroxide solution (200 mL:1 M) and heated to 70–80 °C for half an hour, cooled to room temperature, and filtered out with Whatman filter paper. After washing, the residual product is collected, dried completely in a hot oven, mixed with sodium hydroxide and zinc chloride and a small amount of KOH, and stored for one day at room temperature. It is then carbonized in a tubular furnace at 800–900 °C under atmospheric nitrogen for four hours. This carbonized product is washed with hydrochloric acid (0.1M) until the pH is neutral. The final product is dried and used as a nanocarbon [103].

### 3.4. From Date Palm

Raw material consisting of the date palm is cleaned with deionized H_2_O to remove dust materials, dried at 100–110 °C for 2h, and then mixed with an appropriate activator at a particular ratio for the chemical activation process. This material is boiled at 600 to 800 °C in a graphite furnace for 1 h in an N_2_ atmosphere and the resultant products are cooled at 25–30 °C (pH~7) and washed with distilled H_2_O. The product is dehydrated at 100–110 °C for 2–3 h [104].

### 3.5. From Nicotine Tabacum Stems

**Figure 5:**
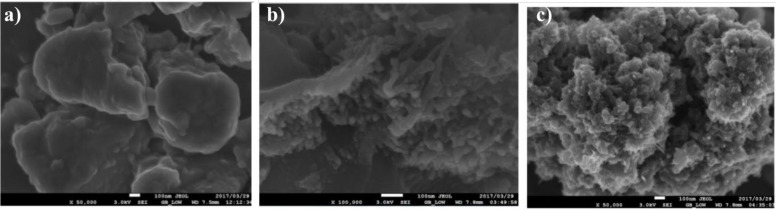
SEM images for nanocarbons carbonized at 400 °C for different time intervals (**a**) 2 h, (**b**) 3 h, and (**c**) 4 h. Adapted with permission from Ref. [105].

**Figure 6:**
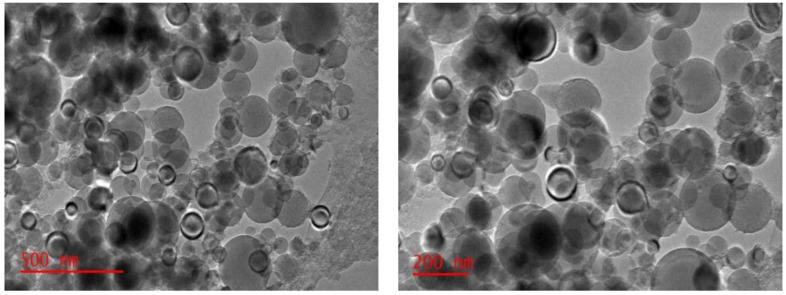
TEM images for nanocarbons carbonized at 400 °C for 4 h. Adapted with permission from Ref. [105].

Due to increasing industrial demand, researchers have focused on recycling and reusing agricultural wastes for the production of valuable products, such as the production of carbonaceous nano-structured materials from bio-waste. Collected tabacum stems are cut into small pieces, dried at 105 °C for 3 days, ground in a mechanical blender, sieved into small pieces, and impregnated with 40 mL of 10% KOH for one day. Then the compound was placed in a muffle furnace under an inert atmosphere, cooled to room temperature and rinsed with 0.1 M HCl and with deionized H_2_O until the pH was 6–5 and dried in an oven at 105 °C [105]. SEM and TEM images of nanocarbons carbonized at 400 ℃ for different time intervals are shown in Figure 5 and Figure 6.

### 3.6. From Various Types of Seeds

#### 3.6.1. From Lapsi Seed Stone

**Figure 7:**
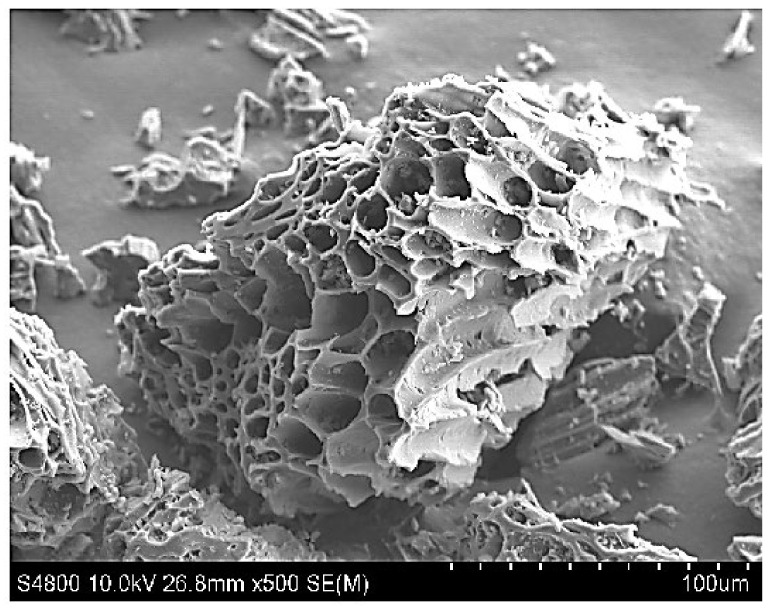
SEM image of highly porous characteristics of activated carbon full of cavities. Adapted with permission from Ref. [107].

Lapsi seeds were collected and washed 3 to 4 times and dried at 110 °C for one day. The crushed powdered form of lapsi seed was mixed with 20 g of KOH and then 100 mL deionized H_2_O was added in a magnetic stirrer at 70 °C for 24 h. The mix is carbonized and dried at 110 °C for 24 h in an electric furnace at 400–500 °C in an inert atmosphere for three hours. The alkaline-treated nanocarbon is further treated with 1N HCl and washed with distilled water until the pH was neutralized. After that, the carbonized compound is dried at 110 °C for 24 hrs and sieved to get a particle size of 212 μm (Figure 7) [106,107,108,109].

#### 3.6.2. From Rubber-Seed Shell

A rubber-seed shell is an alternative and valuable source for the preparation of nanocarbon. These seeds are collected and rinsed with hot distilled water and then dried in an oven at 70–90 °C for one day to give small fine pieces which are activated through a carbonization process with potassium hydroxide for one night. The resulting blackish smooth fine powder obtained after carbonization is collected, washed with cool water, and dried in an oven at 70–80 °C [110,111,112]. 

### 3.7. From Coconut Shell

**Figure 8:**
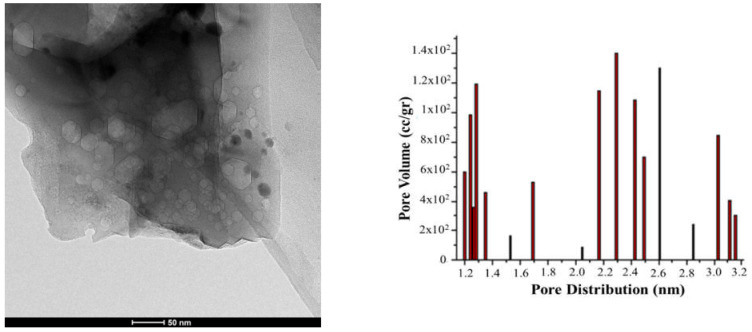
Greater absorption as the temperature rises and TEM Activated Carbon at 700 °C and its pore distribution. Adapted with permission from Ref. [113].

The coconut shell is also a major source of nanocarbon that is prepared by physical and chemical methods. The collected raw material is washed with deionized H_2_O several times in order to minimize the dissolve impurities, dried in the presence of sunlight for 3–4 days, ground to fine particles in mechanical grinders, and then kept in a muffle furnace at 800–1000 °C for 4–6 h by using activated reagent KOH. The impregnated compound is heated in the oven at 90 °C for 3–4 h in the presence of an inert atmosphere. The prepared material is neutralized with KOH until the complete pH is 6–7. The sample is dried in an oven at 80–100 °C for 3–4 h [60,113,114]. TEM images of the activated Carbon at 700 °C and its pore distribution are shown in Figure 8.

### 3.8. From Sugarcane Bagasse

**Figure 9:**
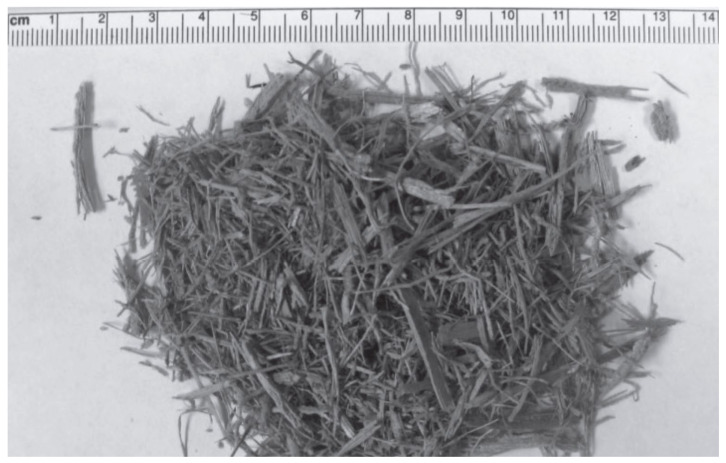
Sugarcane bagasse as received from the ethanol producing company. Adapted with permission from Ref. [115].

**Figure 10:**
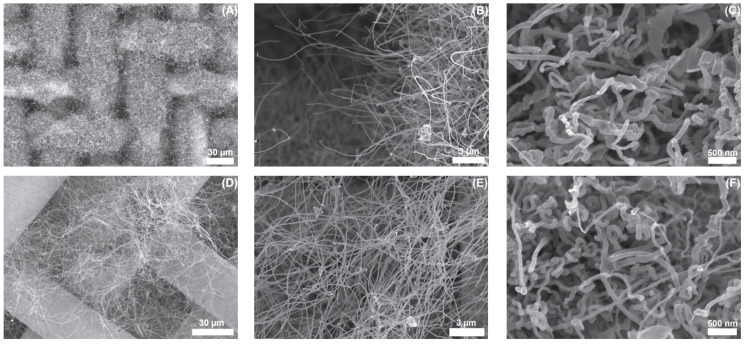
SEM images of sugarcane bagasse and pyrolysis temperatures of: 600 °C (**A**–**C**); and 1000 °C (**D**–**F**). Adapted with permission from Ref. [115].

**Figure 11:**
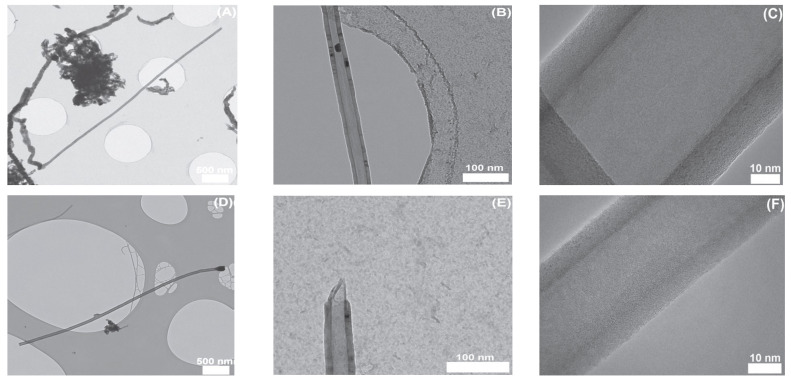
TEM images of sugarcane bagasse and pyrolysis temperatures of: 600 °C (**A**–**C**); and 1000 °C (**D**–**F**). Adapted with permission from Ref. [115].

The raw material is collected in the form of fiber, cut into small pieces in a blender, impregnated into an activating reagent, washed with deionized water, and then converted to nano-materials using the gases generated during the pyrolysis of sugarcane bagasse in the range of 600–1000 °C [115]. Sugarcane bagasse as received from the ethanol-producing company is shown in Figure 9, and SEM and TEM images of sugarcane bagasse are represented in Figure 10 and Figure 11, respectively.

### 3.9. From Nicotiana Tabacum Stems

Nanocarbon is also prepared from green recyclable waste N. tabacum stem using the carbonization process. It is collected and cut into small pieces in a mechanical blender, rinsed with deionized water, and dried at 105 °C for 3–4 days. In a crucible, 10–20 g of dried sample is impregnated into 30–40 mL 10% potassium hydroxide overnight. Then it is placed in a muffle furnace in an inert N_2_ atmosphere, cooled to room temperature by using desiccators, rinsed with 0.1 M HCl, and washed with cooled deionized water until a pH of ~6.5 is attained. The samples are finally dried in an oven at 105 ℃ before they are characterized [105].

### 3.10. From Orange Peel

Nanocarbon is obtained from orange peel by using pyrolysis and pre-carbonization process. For this purpose, orange peel is collected and activated by activating reagent potassium hydroxide at 600–800 °C according to the following chemical reaction.

6KOH + C → 2K + 3H_2_ + 2K_2_CO_3_

**Figure 12:**
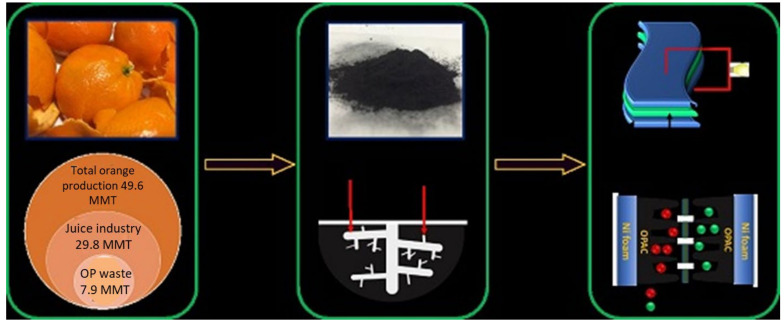
Orange peel by using pyrolysis and pre-carbonization process. Adapted with permission from Ref. [116].

**Figure 13:**
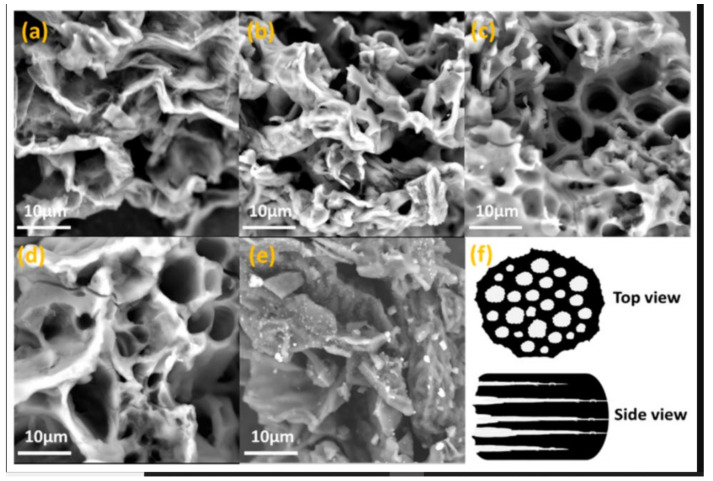
SEM images of (**a**) OPAC-0.5, (**b**) OPAC-1, (**c**) OPAC-2, (**d**) OPAC-3, (**e**) OPUAC, (**f**) schematic diagram of the porous structure in carbon derived from OP. Adapted with permission from Ref. [116].

Consequently, K_2_CO_3_ is decomposed, and the resultant products are further reacted with the carbon to form hollow channels inside the carbon matrix [116]. SEM images of orange peel by using pyrolysis and pre-carbonization process are represented in Figure 12 and Figure 13, respectively.

**Figure 14:**
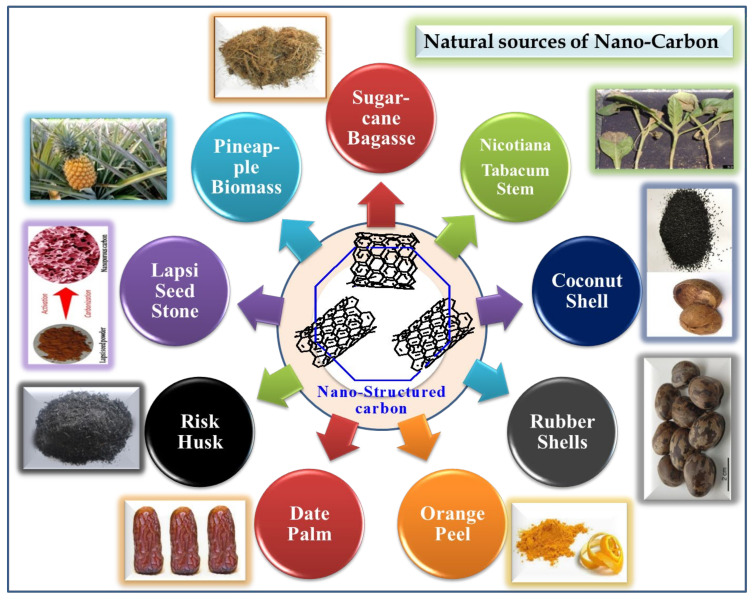
Natural sources of nanocarbon materials from different materials.

All these natural sources of nanocarbon materials from different materials are shown in Figure 14. Detailed investigation of the agricultural waste, the method of synthesis, and the formation of its nanocarbon structure are tabulated in Table 1.

## 4. Application

### 4.1. Energy Storage Applications (Used in Battery, Supercapacitor, and Fuel Cell)

**Figure 15:**
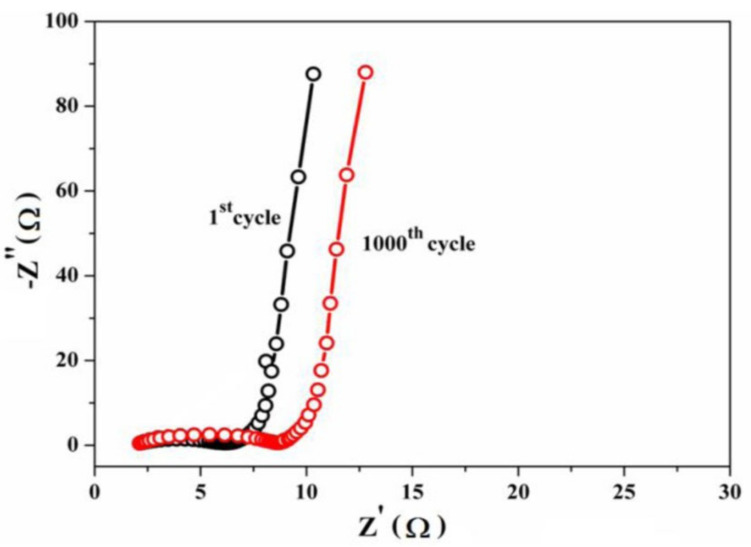
Nyquist plots of the PALF-AC electrode in the frequency ranging from 100 kHz to 10 mHz at open-circuit voltage with an AC perturbation of 10 mV. The EIS measurements were recorded during the cycle stability testing. Adapted with permission from Ref. [117].

Recent advances in the field of nano-batteries are newly designed nanoscale fabricated batteries with particles size less than 100 nanometers or 10^−7^ m. For the battery fabrication, nano-structured carbon is used as an electrode and with carbon nanomaterials that enhance the surface activity of the electrode, increase the current storage of the battery, and decrease the time required to recharge the battery. More current passes between the electrode and the chemical substance within the battery because of the availability of a high surface area. Agriculture waste-derived carbon is used for high-performance lithium-ion capacitors because industrial supercapacitors need high energy demands of graphene-based materials [59]. These graphene-based materials are used as the cathode and LTO (Li_4_Ti_5_O_12_) acts as the anode. This nanocarbon derived from agricultural waste has attracted considerable attention as it offers ease of access, low cost, high chemical inertness, non-toxicity, and high surface area. Bio-plagiaristic carbon concert as supercapacitor electrodes derived from palm leaf waste are used as electrode materials [104]. In addition, fabrication with heteroatoms increases the electrochemical activities and electronic conductivity, leading to enhanced electrode performance [30]. Nanocarbon derived from pineapple leaf by using chemical activating agents such as KOH is used as electrochemical capacitors (ECs) or supercapacitors (SCs) as energy storage devices that can be potentially combined with renewable energy technology. SCs devices are used in long-life power storage capacitors with a higher power density (Figure 15) [117]. Carbon dots (CDs) are an example of nanocarbon. After toughening, they possess excellent conductivity due to their crystalline property, which improves the mobility of electrons, and the thermal stability of such design is also useful in lithium-ion batteries [9]. Fabrication of lithium-ion batteries with some metal oxide such as CuO/Cu and CuO/Cu enhances the transport kinetics of electrons because of the good electrical conducting nature of copper and graphene carbon atoms. The excellent chemical, mechanical, electrical, and thermal properties of these carbon nanostructures have led to applications in many energy storage and conversion devices. The importance of carbon nanomaterials in the large-scale energy sight is a topic that has attracted great interest in modern times [59,118]. It is a widespread material, and fossil fuels are environmentally eco-friendly and usually nonrenewable. Therefore, supercapacitors as fuel cells are electrochemical devices that are used as energy storage devices. Chemical energy converted into electrical energy by the fuel cell is considered safe and does not produce any destructive gases (normally greenhouse gases).

### 4.2. Used in Polymer Nano-Composite Materials

Several nanocomposites and nanocoated polymers are used in the automotive and solar energy fields [119]. According to the literature over the last few decades, nanocarbon composite polymer has significant quality and unique properties for various applications such as in biosensors, drug release, and antimicrobial activities. These compounds have high electrical and optical thermal properties [120,121]. Carbon-based nanomaterials exhibit diverse applications along with environmental remediation, sensors, drug launch antibacterial reagents, crop safety, and growth regulator of the plant due to particular characteristics such as optical, electrical, mechanical, and thermal quality. The synthesis of carbon primarily based on nanomaterials has received giant investments including activated carbon, activated carbon fibers, CNTs, CNFs, graphene, and fullerenes, which have the largest interest in agriculture. Substantial research has been conducted on carbon-based nanomaterials, including CNTs, CNFs, and graphene synthesized from various secondary agricultural sources. Among these nanocarbon CNS, CNFs has excellent capture ability as well as release in balancing manner of metals nanoparticles such as Cu, Zn, and Fe. Therefore, CNFs are a good transporter of micronutrients [122]. 

### 4.3. Used in Biosensors

Biosensors are an analytical tool with significant application in the identification of biological compounds such as an advanced technique for sensing ions, organic catalysts (enzyme), antibodies, some modified biological compounds (proteins, antibodies), and heavy metals in water. The transducer undergoes physiochemical changes in the form of the current [121]. In the biological field, different kinds of transducers are used to identify the biosensor, reduce the activation energy, and increase the catalytic power so it can be catalytic or a similarity-based source. Nanocarbon tubes are a potential material with a greater affinity towards the modified biosensor. Nanocarbon-based biosensor modified by glycan has been used for the identification of influenza virus [123,124]. Nanocarbon resources have huge applications in the latest devices because of their high surface area and high mobility. Modified fabricated nanocarbon is used as a pH sensor which shows different sensing values with ion concentration. Nanocarbon is valuable in the biomedical and biosensor fields because it has good optical, electrical, and thermal stability qualities [125]. Small-sized proteins are adsorbed very smoothly on the surface of nanocarbon and are used for gene release because they have high resistance to enzymes. It has direction ability towards nucleic acid (DNA, RNA) by self-assembly involves intercellular translocation and is also used in drug delivery systems and in cancer treatment. Enzymatic sensitive electrochemical-based carbon nano compound modified with various metal oxides is used as a sensor [126]. The different nanostructures of the carbon give advanced ideas that can modernize the approach for gas sensors, electrochemical sensors, and biosensors, which are the best examples of modified carbon compounds. Carbon in nano form has the greatest capability for multi-functional sensing applications due to its distinctive high electrical and chemical qualities, biocompatibility, and ease of fabrication from various kinds of inorganic nanostructure materials and biomolecules [127,128,129,130]. Cyclic voltammetry and chronoamperometry studies of nanostructured carbon fabricated with Bi_2_O_3_NPs and AuNps show better sensing performance and lower potential compared to others [125,131]. A biosensor device consisting of carbon nano form has unique bio-inspired receptor links towards the biological-analyst such as the DNA of bacteria or viruses and protein, which is generated through infected living organisms [132].

### 4.4. Used in Water Treatment

**Figure 16:**
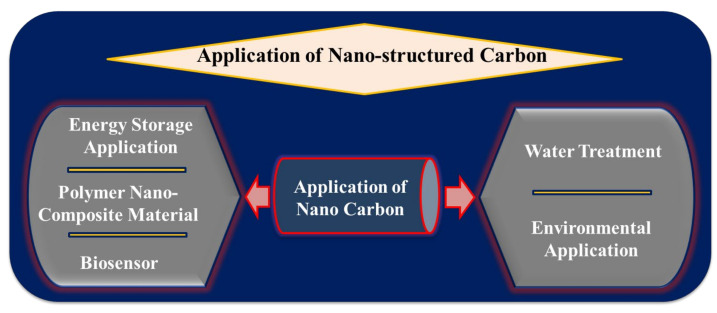
Pictorial description of nano-structured carbon applications in different forms.

Today the world is facing the biggest problem of polluted water. Without water, life is not possible because water is life. On Earth, only 1% of water is available for drinking and a great deal of water has been polluted through human activities. The major sources of water pollution are leakage of raw material from industries and toxic industrial waste, biomedical, agricultural waste, and some toxic chemical contaminants such as micro-pollutants, organic and inorganic pesticides, and various types of water-soluble dyes. The conversion of wastewater into drinking water and its purification requires the development of advanced nanomaterials, which are easily available from natural sources. Therefore, we need to reduce utilization and focus on the development and protection of our water resources. The conventional processes for the treatment of polluted water to drinking water need sufficient and capable nanomaterial. For wastewater treatment, nanotechnology offers possibilities for the efficient elimination of pollutants and microorganisms [133]. Nanocarbon materials are used for the removal of heavy metals from water, which is an excellent approach as it is very methodical and allows trace levels of heavy metal ions. Carbon nanomaterials such as fullerene, carbon nanotubes (CNTs), graphene, modified graphene, and other nano forms of carbon are used in the purification of heavy metal ion-contaminated water [134,135]. Nanocarbon is easily functionalized with various substituted organic molecules that could make them unique for the selection of adsorbates and their adsorption capability might be upgraded. Because of its minute size and resulting high specific surface area, nanomaterials have high adsorption power and reactivity. One of the most popular forms of carbon is CNTs, which have attracted much attention due to their distinctive qualities. They have large porous structures and high adsorption ability and therefore can adsorb many organic molecules and ions, including dichlorobenzene, ethylbenzene, Zn^2+^, Pb^2+^, Cd^2+^, Cu^2+^, and some other dyes. Both multi-walled and single-walled carbon nanostructures are used for this purpose [135,136,137]. To enhance the adsorption, chemical, optical, and electronic qualities of nanocarbons functionalized with heteroatoms such as oxygen, nitrogen, and other metal oxides, these modulated nanocarbons are used for dyes removal such as methyl violet from water. Methylene blue is removed by electrochemical fictionalization of the nanocarbon surface by the π-stacking interaction between the aromatic compounds [137,138]. The overall descriptions of nano-structured carbon applications in different forms are shown pictorially in Figure 16.

## 5. Conclusions

Useful products made from waste materials are essential nowadays to minimize environmental waste and pollution. Carbon nanomaterials are used in various fields, including water treatment, environmental applications, energy storage and conversion, and as biosensors in the field of biomedicine. To prepare these versatile carbon materials, different methods are used, and their structures can be tuned. Simple carbonization, microwave method, hydrothermal method, and some hybrid methods can be used for the synthesis of carbon nanomaterials. This review has presented an overview of the literature describing the methods for preparing useful products by utilizing agriculture-based waste materials such as sugar cane bagasse, rice husk, pineapple peels, coconut shells, and tobacco stalks. This review gives the direction of the usage of renewable sources to achieve the best materials, even in large quantities. Utilization of waste resources leads to reducing the consumption of natural sources such as storage applications, water treatment, and so on. These can have more advantages in the near future since the artificial (unnatural) sources are limited, which needs an ultimate alternative for future generations.

## Figures and Tables

**Table 1:** Detailed investigation of the agricultural waste, method of synthesis, and its carbon structured formation.

S. No	Agricultural Waste	Method of Synthesis	Carbon Structured Formed	Reference
1	Sugar cane	By pyrolysis	Carbon nanotube	100
2	Pine apple	By hydrothermal	3D continuous network of homogeneous micro/mesopore structure	105
3	Rice husk	By microwave	Carbon nano tube	82
4	Date palm	By hydrothermal	Porous carbon	104
5	Nicotine tabacum stems	By carbonization	Nanocarbons	101
6	Lapsi seeds, Rubber-seeds	By chemical activation	Honey comb structure	91
7	Coconut shells	By pyrolysis methods	Mesoporous	97
8	Orange peels	Chemical activation as well as pyrolysis	Sheet-like structure	102

## Data Availability

Not applicable.
